# Evaluation of an internet-based aftercare program to improve vocational reintegration after inpatient medical rehabilitation: study protocol for a cluster-randomized controlled trial

**DOI:** 10.1186/1745-6215-14-26

**Published:** 2013-01-25

**Authors:** Rüdiger Zwerenz, Katharina Gerzymisch, Jens Edinger, Martin Holme, Rudolf J Knickenberg, Sieglinde Spörl-Dönch, Ulrich Kiwus, Manfred E Beutel

**Affiliations:** 1 Department of Psychosomatic Medicine and Psychotherapy, University Medical Centre, Johannes Gutenberg-University, Mainz, Germany; 2 German Statutory Pension Insurance Rehabilitation Centre for Orthopaedics, Clinic Weser, Bad Pyrmont, Germany; 3 Clinic for Psychosomatic Rehabilitation, Rhön-Klinikum AG, Bad Neustadt/Saale, Germany; 4 Clinic for Prevention and Rehabilitation of Cardiovascular Diseases, Haus Franken GmbH, Bad Neustadt/Saale, Germany; 5 German Statutory Pension Insurance Rehabilitation Centre for Cardiovascular Diseases, Clinic Wetterau, Bad Nauheim, Germany

**Keywords:** Internet-based intervention, Medical rehabilitation, Premature pension, Rehabilitation aftercare, Stress management, Work disability, Work stress

## Abstract

**Background:**

Mental disorders are the main reasons for rising proportions of premature pension in most high-income countries. Although inpatient medical rehabilitation has increasingly targeted work-related stress, there is still a lack of studies on the transfer of work-specific interventions into work contexts. Therefore, we plan to evaluate an online aftercare program aiming to improve vocational reintegration after medical rehabilitation.

**Methods:**

Vocationally strained patients (n = 800) aged between 18 and 59 years with private internet access are recruited in psychosomatic, orthopedic and cardiovascular rehabilitation clinics in Germany. During inpatient rehabilitation, participants in stress management group training are cluster-randomized to the intervention or control group. The intervention group (n = 400) is offered an internet-based aftercare with weekly writing tasks and therapeutic feedback, a patient forum, a self-test and relaxation exercises. The control group (n = 400) obtains regular e-mail reminders with links to publicly accessible information about stress management and coping. Assessments are conducted at the beginning of inpatient rehabilitation, the end of inpatient rehabilitation, the end of aftercare, and 9 months later. The primary outcome is a risk score for premature pension, measured by a screening questionnaire at follow-up. Secondary outcome measures include level of vocational stress, physical and mental health, and work capacity at follow-up.

**Discussion:**

We expect the intervention group to stabilize the improvements achieved during inpatient rehabilitation concerning stress management and coping, resulting in an improved vocational reintegration. The study protocol demonstrates the features of internet-based aftercare in rehabilitation.

**Trial registration:**

International Standard Randomised Controlled Trial Number Register (ISRCTN:ISRCTN33957202)

## Background

Sick leave and premature pension are rising constantly in most high-income countries because of mental disorders [1-3]. Job strain has been proven to be an important determinant for mental and somatic disorders [4,5]. Approximately one third of the patients in medical rehabilitation are reporting significant work-related stress in Germany [6-8]. Therefore, various job-related interventions have been adopted during inpatient treatment to improve vocational reintegration after rehabilitation [9,10]. Whereas the positive effects of job-related interventions on treatment satisfaction and the intention to return to work could be confirmed in short-term analyses [11-15], transfer to patients’ daily work remains to be demonstrated, especially following prolonged work disability [16,17]. New stressors created by rapidly changing tasks, procedures and technologies, and also interpersonal conflicts in the workplace, may lead to a relapse of symptoms and, finally, to an incapacity for work or premature pension. Only by considerable efforts of networking and implementing aftercare programs does the sustainability of treatment effects seem achievable [18,19]. Currently, only a limited number of patients are assumed to have access and to use immediate aftercare following inpatient medical rehabilitation [20]. Sibold and colleagues [21] reported that the most frequently given reasons for non-participation in aftercare programs included incompatibility with duties at work (70.7%), too much time investment (46.5%) and poor access to the outpatient rehabilitation facility (34.1%). Accordingly, travelling time proved to be a significant negative predictor for participation.

Several controlled trials demonstrated the efficacy of internet-based therapy and counseling for various mental disorders [22]. In Germany, 92% of the 30- to 49-year-old age group and 69.1% of the 50- to 59-year-old age group have private web access [23]. The results of Kobelt *et al.*[24] indicate the acceptance of internet-based assessment and diagnostics in inpatient medical rehabilitation. Thus, online interventions are not only more easily accessible for far more patients than outpatient treatment [25] but are also more cost effective [26,27]. Although recent evaluation studies on internet-based aftercare programs after inpatient rehabilitation indicate high patient satisfaction, acceptance and efficacy [28-31], the current percentage of online interventions in rehabilitation amongst all internet-based intervention programs is still very low, with a 9% share [32].

Writing is an important component in most online aftercare programs. Based on Pennebaker’s paradigm [33], the healing effects of expressive writing on both the mental and physical state have been widely accepted [34-38]. Direct feedback by the therapist and by fellow patients presumably has an additional motivating and encouraging function and may also initiate model learning [39]. In a meta-analysis comparing different self-care interventions for chronic disease, Wantland and colleagues [40] found that the participation rate in web-based interventions is higher in interventions that are individually tailored compared with less individual interventions.

To promote the transfer of vocational stress management from inpatient treatment to the work setting, we devised an internet-based aftercare intervention with a weekly expressive writing task, followed by individual feedback from an online therapist within one week day and a moderated patient forum. We compare this individually tailored and therapist-moderated ‘active synchronous intervention’ [41] to a passive (control) intervention by means of an informational website, where patients are regularly alerted by e-mail to publicly accessible information concerning stress management and coping. The online aftercare program extends a manualized vocational stress management group training (Gesundheitstraining Stressbewältigung am Arbeitsplatz; GSA) administered during inpatient rehabilitation through to aftercare. The internet-based aftercare intervention also includes audio samples with relaxation exercises and the GSA worksheets. The efficacy of the GSA training has been demonstrated in short-term analyses for patients treated in psychosomatic, cardiovascular and orthopedic inpatient rehabilitation centers [42], and half of the cooperating clinics have been using parts of the GSA manual since 2006 in their regular stress management programs. With the design of our online aftercare program, we aim to close the gap between inpatient treatment and aftercare and evaluate the acceptance and efficacy of the program across different indications of medical rehabilitation. We expect that participating in online aftercare will lead to a reduced risk of premature pension assessed with a standardized and validated questionnaire.

## Methods

### Participants

Patients are recruited during inpatient psychosomatic, orthopedic or cardiovascular rehabilitation. Eligible patients have to be vocationally strained, assessed by the ‘Screening Instrument zur Messung des Bedarfs an berufsbezogenen Behandlungsangeboten in der medizinischen Rehabilitation’ (Short Screening Instrument for the Assessment of Need for Occupation Related Treatment in Medical Rehabilitation; SIBAR [6]), a short screening questionnaire measuring the social medical risk of early retirement, occupational stress and the subjective need for occupational treatment. Patients are eligible if they report high occupational stress or risk of early retirement appropriate to the cutoff reported by the authors of the SIBAR or if they state a high subjective need for occupational treatment. Furthermore, eligible patients have to be German speaking, currently employed, between 18 and 59 years old and have private internet access, so that they do not have to use internet access at work for participation in our study and are not disturbed by others. Patients are excluded if they are unemployed or retired, or have severe physical or psychological complaints, precluding participation in the stress management training (GSA). We also excluded anyone aged over 59 because the likelihood of retirement is very high over 59 years, limiting therapeutic access to these patients. The Study Centre of Mental Disorders at the University Medical Centre of the Johannes Gutenberg University of Mainz is responsible for storing personal data, encoding the participants and randomizing the groups. Administration of the internet platform, allocation of the weekly writing tasks and therapeutic feedback are managed by psychologists of the Department for Psychosomatic Medicine and Psychotherapy of the University Medial Centre Mainz. The clinical staff of the four rehabilitation centers (one psychosomatic, one orthopedic and two cardiovascular) conducting the inpatient GSA program and introducing the internet-based aftercare to the patients consists of social education workers, psychologists and psychotherapists, all experienced in vocational stress management and psychological group training.

Data security is guaranteed by secure sockets layer-coded internet connections as used in bank transfers and a firewall-protected webserver for both the MySQL-database and the application of the internet platform. All patients are instructed to use pseudonyms to login and for all actions on the internet platform so that no personal data is stored on the webserver; therefore, identification of the user is not possible.

Over a period of 18 months, a total of 800 patients is planned to be included in the study (100 rehabilitants in each center for each randomized arm).

### Interventions

At inpatient admission, patients are informed in detail about the study by the clinical staff of the rehabilitation centers. After giving written consent and screening, the eligible patients are assembled into a group of up to 12 (minimum of two) participants. Over a period of two to three weeks, participants are trained in stress perception and management by four interactive psycho-educative, cognitive-behavioral and psychodynamic modules (90 minutes each). To standardize the procedure, the staff has been trained using a comprehensive manual in a detailed one-day seminar before study start, with a refresher half-way through recruitment. In the first part of the fourth module, the participants are familiarized with the three components of Luborsky’s Core Conflictual Relationship Theme and its recurrent and potentially mal-adaptive nature, focusing on typical expectations regarding return to work [43,44]. This approach also serves as a basis for the upcoming writing task in the intervention group. The introduction to the features of the internet platform, given in the last of four inpatient GSA modules, differs between the two randomized arms. Up to the fourth session, participants and trainers are blind to the result of randomization. Immediately prior to the fourth module, the trainer is unblinded. In the second part of the fourth session, all participants are introduced to the special features of the internet platform. Instructions for participants depend on the arm they are randomized to (intervention versus control). Because there is plenty of interaction between consecutive cohorts of patients admitted to medical rehabilitation, we do not randomize individuals but groups of eligible patients. With this cluster randomization, we aim to minimize contamination through the interaction of patients during inpatient rehabilitation who are getting different instructions for the aftercare interventions in the two randomized arms.

In two of the four rehabilitation centers, patients of both randomized arms have the opportunity to log on to the study website during their inpatient rehabilitation treatment and complete the baseline questionnaire online. In the other two clinics without sufficient technical equipment, the participants get the same questionnaire on a paper-pencil basis and are prepared for their first login at home by the trainer. In any case, every participant gets a sealed envelope including the personal access data and a written description of the intervention or control program. After login, all patients are asked to change their password, to choose a nickname and to enter their e-mail address if they want to be informed by automated e-mails about study-related information, therapeutic tasks and questionnaires to be filled out.

The essential component of the online aftercare program for the intervention group is a standardized weekly writing task (‘blog’) followed by individual feedback from the online therapist within one week day, both only visible to the individual patient and the online therapist. Further online features for the intervention group are a self-test with computer-generated feedback to the participants’ individual ‘Arbeitsbezogenes Verhaltens und Erlebensmuster’, Kurzform 44 (Pattern of Work-related Coping Behavior, short-form 44; AVEM-44 [45]) and its change over time, audio samples with progressive muscle relaxation exercises [46], the GSA worksheets and a moderated patient forum. All these features are accessible for 12 weeks. The internet-based therapeutic interventions are performed by two trained psychologists with regular supervision. With the participants’ consent, all blog contacts between therapist and patient are fully documented in the database, allowing subsequent comparisons and analyses concerning the contents.

The control group receives regular e-mail reminders to use selected and online-deposited information about stress management and coping (physical activity, relaxation, healthy diet and sleep hygiene) over the same period of time. All information is permanently downloadable for participants of the control group for the duration of 12 weeks. Overall there are six e-mail reminders, that is, one every fortnight, beginning with a universal reminder, followed by reminders for the five specific topics on stress management and coping.

### Assessment

**Figure 1:**
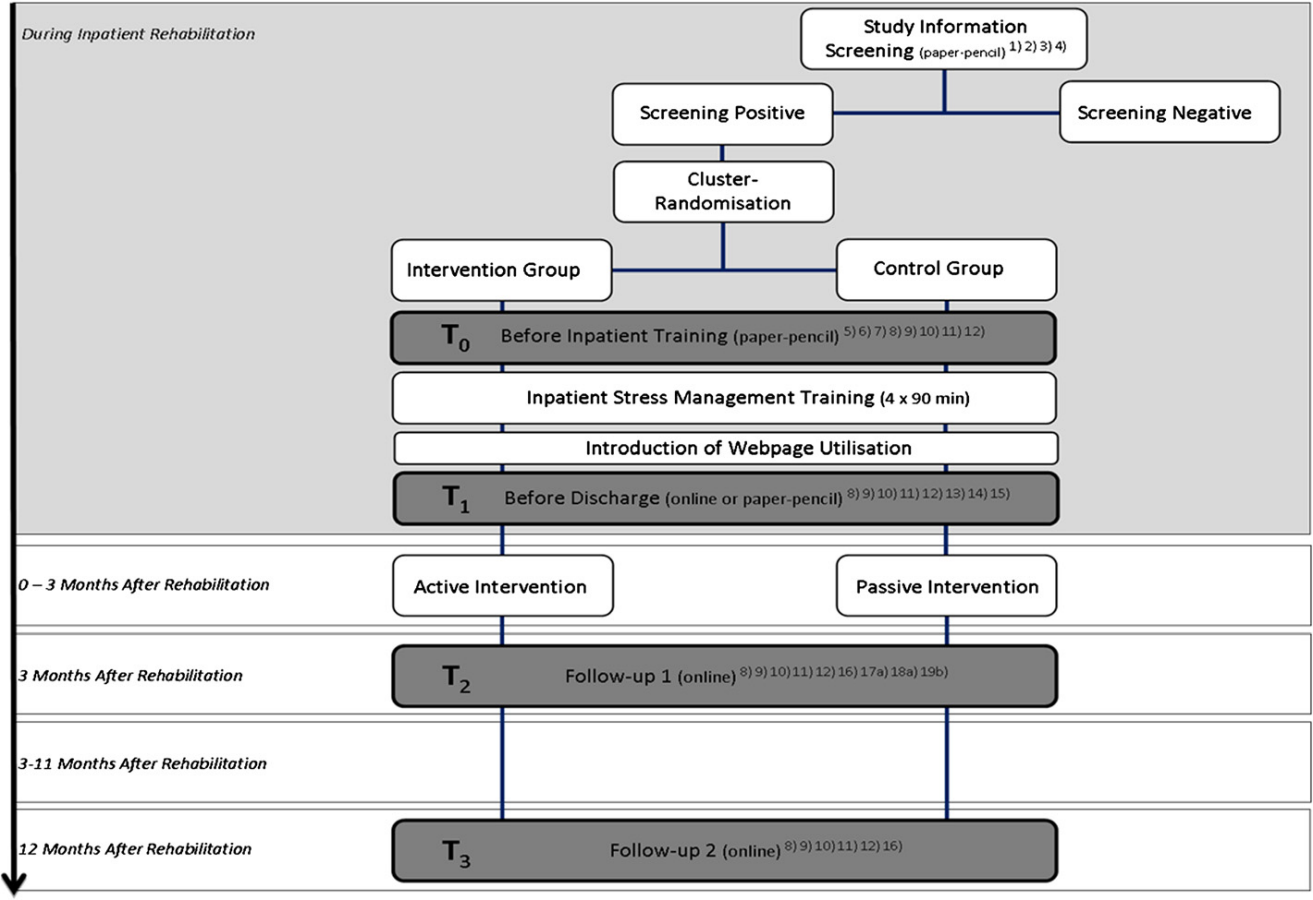
**Study design and time points of assessment.**^1) ^patient information, ^2) ^declaration of consent, ^3)^ Screening instrument work and occupation screening, ^4) ^check of inclusion criteria, ^5) ^socio-demographic data, ^6) ^previous treatment, ^7)^ internet use, ^8) ^Arbeitsbezogenes Verhaltens und Erlebensmuster, Kurzform 44 (Pattern of Work-related Coping Behavior, short-form 44), ^9)^ Patient Health Questionnaire, ^10) ^Generalized Anxiety Disorder 7-item Scale, ^11) ^Short-Form 12-item Health Survey, ^12) ^Berlin Social Support Scale, ^13) ^Fragebogen zur Messung der Patientenzufriedenheit (Questionnaire on Patient Satisfaction), ^14) ^inpatient treatment satisfaction, ^15)^ medical discharge report, ^16) ^further treatment, ^17a) ^Helping Alliance Questionnaire (only intervention group), ^18a) ^Helping Alliance Questionnaire II (only intervention group), ^19b) ^frequency of use and satisfaction with website content (different for the intervention and control group).

As shown in Figure 1, the time points of assessment by self-rating scales are at the beginning (T0) and the end (T1) of inpatient rehabilitation, at the end of the aftercare (T2 = 3 months after T1) and 9 months later (T3 = 12 months after T1). Additional somatic and psychological measures, documented during inpatient rehabilitation, are assessed at T1.

Depending on the technical equipment in the rehabilitation centers, T1 assessment is conducted online or paper-pencil. All following data are collected internet based, unless the patients do not participate within two weeks. In this case, the patients get paper-pencil questionnaires by mail. The online assessment takes about 45 minutes, comparable to the paper-pencil assessment. Patients get computer-generated feedback following each completed online questionnaire, describing the participants’ individual work-related behavior and experience pattern and its change over time, measured by the AVEM-44 [45]. The feedback is intended to serve as an incentive for participating in the survey.

### Objectives and hypotheses

In this study we evaluate the acceptance and efficacy of an internet-based aftercare program for three different indications of medical rehabilitation (at the participant level), with the following hypotheses:

1. We expect that taking part in the internet-based aftercare program helps stabilize the improvements achieved during inpatient rehabilitation concerning stress management and coping with conflicts at the workplace. Therefore, participants of the intervention will have a lower risk for premature pension than the control group.

2. We expect these effects to persist up to 9 months after the end of the internet-based treatment.

### Outcomes

The primary outcome measure is a risk factor for early pension, measured by the screening questionnaire SIBAR, a score ≤8 indicating a decreased risk for premature pension. The primary endpoint will be the assessment at the end of the internet-based treatment (T2, that is, 3 months after inpatient rehabilitation). Secondary outcome measures include the subjective prognosis for work and capability, also measured by the SIBAR, and scales for the work-related behavior and experience pattern (AVEM-44 [45]), physical and mental health (Patient Health Questionnaire [47], Generalized Anxiety Disorder 7-item Scale [48], Short-Form 12-item Health Survey [49]), social support (Berlin Social Support Scale [50]), therapeutic alliance (Helping Alliance Questionnaire [51], Helping Alliance Questionnaire II [52]) and self-developed items measuring patients‘ satisfaction with the aftercare program. Long-term effects of the primary outcome measure will be analyzed 9 months after termination of the internet-based aftercare at T3. Except for the Helping Alliance Questionnaires, only applied in the intervention group, all instruments are used in both randomized arms.

### Sample size calculation

At present, there are no reports of reliable effect sizes for internet-based interventions aiming at the improvement of vocational strains through online aftercare interventions. We therefore made conservative calculations with an expected small to moderate effect size (d = 0.30), a statistical power of 0.80 and alpha of 0.05. A total sample size of n = 190 will be necessary to gain significant results (calculation with GPower 3.0.10 for analysis of variance with fixed effects, special, main effects and interactions).

Patients are randomized in clusters - the cluster size is set at two to twelve participants. For each clinic, 30 blocks have been generated, each consisting of both groups in random order. Altogether, there is a maximum of 60 clusters per clinic with an equal share of intervention and control groups. To adjust sample size calculation for clustering, we multiplied the needed sample size by the design effect (1+(m-1)ρ, with m = average cluster size and ρ = intracluster correlation coefficient) as suggested in the CONSORT statement extension for cluster randomized trials [53]. With an anticipated average cluster size of six participants and an intracluster correlation coefficient of ρ = 0.30, as reported by Campbell *et al*. for comparable studies [54], we calculated a design effect of 2.5. By multiplying our needed sample size of n = 190 with the design effect we get a minimum sample size of n = 475 needed for robust statistical analyses. Even in case of a dropout rate of 40% as reported in a recent aftercare study [21], the anticipated number of n = 800 (n = 100 per clinic and condition) will provide statistically confirmed data.

### Randomization

With the help of the computer software Research Randomizer [55], a block randomization has been conducted by the Study Centre of Mental Disorders. Stratified according to the four clinics to achieve an equal share of intervention and control groups, the lists have been assigned to the trial sites by a researcher not involved in the study.

### Statistical methods

For the primary analyses, analysis of covariance will be used to compare SIBAR risk scores 12 weeks after the discharge of inpatient rehabilitation (T2) between the internet-based aftercare program (intervention group) and the control group, with covariates for rehabilitation clinic and baseline SIBAR risk score (T1). Cohen’s effect sizes will be calculated. Intention to treat analyses as well as completer analyses will be conducted. Replacement strategies of missing values will be discussed after assessing the pattern of the missing value structure.

For the secondary analyses, self-report questionnaires (AVEM-44; Patient Health Questionnaire; Generalized Anxiety Disorder 7-item Scale; Short-Form 12-item Health Survey; Berlin Social Support Scale; Helping Alliance Questionnaire) will be analyzed by mixed models with repeated measurements. It is expected that the SIBAR risk score and the other self-report measures will not be linear across time. Therefore, these measures will be evaluated by a linear model with fixed effects for treatment and rehabilitation center and time. There will be an indicator variable for the post treatment measurements and the SIBAR risk score at baseline will serve as a covariate. All analyses will be conducted on a two-sided level of significance of α = 0.05.

### Ethical issues

The study protocol and the final version of the written informed consent form were approved by the Ethics Committee of the Federal State of Rhineland Palatinate (Germany), which is responsible for the Principal Investigator (Ref. No. 837.415.10[7424]) and by the ethics committees responsible for the cooperating rehabilitation clinics.

The procedures set out in this protocol pertaining to the conduct, evaluation and documentation of this trial were designed to ensure that all persons involved in the trial abide by Good Clinical Practice and the ethical principles described in the current revision of the Declaration of Helsinki. The trial will be carried out in keeping with local legal and regulatory requirements.

Before being admitted to the clinical trial, patients must consent to participate after the nature, scope and possible consequences of the clinical trial have been explained in a form understandable to them. The patients must give written informed consent to participate in the study, including their consent to publish.

We expect a very low risk for adverse events. Nevertheless, participants have the possibility to contact the staff at the study center via e-mail or on a mobile help number with the guarantee of a response during working hours (8.00 a.m. to 5.00 p.m.) on weekdays. On weekends and at all other times, patients are referred to a Germany-wide crisis telephone number, which is indicated on our internet platform. All adverse events reported by the participants or detected by the online therapist or respective study staff will be collected during the trial and must be documented in the case report form. The clinical course of the adverse event will be followed by the principal investigators (MEB, RZ), who will contact the participants in case of a crisis for further diagnostics via telephone or for necessary referrals to practitioners near the participant.

## Discussion

Work-related stress often leads to serious somatic and psychosomatic complaints and, vice versa, chronic diseases of different indications have a negative impact on job strain. For this complex interdependency, the German rehabilitation system has developed a focus on work-related interventions during inpatient medical rehabilitation [10]. High quality studies have confirmed that work-related medical rehabilitation has favorable effects on earning capacity and work-life participation [56]. Nevertheless, we are still uncertain how patients could transfer these treatment outcomes to their daily workplace. The online aftercare program is expected to provide participants with a temporally and geographically flexible, but also reliable, support. We assume that online interventions are easier to integrate into daily work life than outpatient programs [57] that may be incompatible with duties of work and are difficult to access.

Based on previous studies, we expect good acceptance of internet-based data ascertainment and diagnostics in inpatient medical rehabilitation [24] and a high rate of rehabilitants having private web access. Bearing in mind that the online therapist is not the inpatient therapist but an anonymous person, and that the patients are required to initiate or continue the aftercare program at home on their own, some skepticism and reticence can nevertheless be expected among the participants. It may be suspected that those patients who tend to object to the web-specific anonymity do not benefit at the same level as those who generally appreciate anonymity [57]. One of the main tasks may therefore be to convince the patients of the confidentiality and data security within the study information. The individual benefit may also depend on factors like web accessibility, technical interest, confidence and understanding, which will require sufficient support, training and motivation from the clinical staff. The inpatient clinical staff is experienced in vocational stress management, has been trained in the four GSA modules as well as in presenting the internet platform, and is in a permanent exchange with the study center. Nevertheless, we will not be able to prevent trainer bias completely. A strength of our study is that we have chosen three of the major indications in the German medical rehabilitation system comprising most of the main causes for work disability and premature pension. As established in previous trials with inpatient interventions for psychosomatic, cardiovascular and orthopedic rehabilitation patients, there are comparable rates of vocational problems in these three indications. In the previous work, we could also establish that the inpatient interventions are effective in improving work-related attitudes across these three indications. It remains to be proven if findings are specific for each disease entity or inpatient rehabilitation center. Still, we expect differences between the three categories regarding age, gender, comorbidities, and so on. We therefore plan to take the category of patient into account as an outcome predictor.

One of the other challenges of the study seems to be the lack of immediate interaction, which presumably requires more incentives for the participants than in face-to-face interactions [58]. It remains to be seen whether the computer-generated feedback following each completed online questionnaire in both randomized arms will serve as a sufficient incentive.

One restriction is that not all rehabilitation centers have the resources for online assessments during inpatient rehabilitation, specifically for an introduction to the internet platform. Following the review of Wantland and colleagues [40], we assume the online and paper-pencil collected data vary little in reliability and validity, although bias caused by the different ways of data collection cannot be excluded completely [59,60]. However, we cannot completely preclude that filling out questionnaires online during inpatient rehabilitation may increase participation in our program. We will therefore compare the frequency of utilization between those who fill out baseline questionnaires online and offline. Furthermore, we have to expect that missing out on an introduction to the internet platform in practice during inpatient rehabilitation is leading to a higher rate of patients dropping out of the study. Therefore, the rehabilitation center will be an important covariate in the statistical analyses.

By evaluating our online intervention comprehensively, we aim to estimate the short-term and especially the long-term effects of the program on stress management and coping concerning job strain and, finally, on successful occupational reintegration.

With the cluster randomization, patients are enrolled in the study as cohorts and therefore we are confident that they are more motivated to take part and less likely to drop out of the study than in an individually randomized design. With the, for the most part, blinded inpatient training we try to enable that both intervention group and control group are treated the same during inpatient rehabilitation. Unfortunately, a completely blinded assessment of the outcome was not possible, because patients needed to be informed about the group and intervention they are randomized to, during inpatient rehabilitation. However, our main outcome is assessed by study participants, independent from the online therapist. The fact that the intervention group and control group use the same medium after inpatient rehabilitation for the follow-up assessments will help us to reduce possible confounding factors when interpreting the results of the comparative analyses. Overall, and from a practical point of view, we think that not only because of the close connection between the design of our online aftercare program and the inpatient GSA training during rehabilitation but also because of the interdisciplinary approach, combining cognitive, behavioral and psychodynamic elements in training and aftercare, our internet-based aftercare program could be a very promising supplement for inpatient rehabilitation.

## Trial status

The first patients were enrolled to the study on 1 July 2011. Follow-up assessments for the last included patients are expected to be completed by September 2013.

## Abbreviations

AVEM-44: Arbeitsbezogenes Verhaltens und Erlebensmuster Kurzform 44 (Pattern of Work-related Coping Behavior, short-form 44); GSA: Gesundheitstraining Stressbewältigung am Arbeitsplatz (Health training for stress management at the workplace); SIBAR: Screening Instrument zur Messung des Bedarfs an berufsbezogenen Behandlungsangeboten in der medizinischen Rehabilitation (Short Screening Instrument for the Assessment of Need for Occupation Related Treatment in Medical Rehabilitation.

## Competing interests

The authors declare that they have no competing interests.

## Authors’ contributions

RZ wrote the first draft of the manuscript. RZ, KG, JE and MEB wrote the final draft of the manuscript and critically revised it for its intellectual content. RZ, KG, JE, MH, RJK, SSD, UK and MEB substantially contributed to the conception and the design of the study. All authors read and approved the final manuscript.
